# Efficacy of a Three Drug-Based Therapy for Neuroblastoma in Mice

**DOI:** 10.3390/ijms22136753

**Published:** 2021-06-23

**Authors:** Patrizia Garbati, Raffaella Barbieri, Matilde Calderoni, Francesca Baldini, Mario Nizzari, Paola Modesto, Tullio Florio, Aldo Pagano

**Affiliations:** 1 IRCCS Ospedale Policlinico San Martino, 16132 Genova, Italy; p.r.garbati@gmail.com (P.G.); raffaella.barbieri@edu.unige.it (R.B.); tullio.florio@unige.it (T.F.); 2 Department of Experimental Medicine (DIMES), University of Genova, 16132 Genova, Italy; calderoni.matilde@gmail.com (M.C.); baldinifrancesca92@gmail.com (F.B.); mario.nizzari@unige.it (M.N.); 3 National Reference Center for Veterinary and Comparative Oncology-Veterinary Medical Research Institute for Piemonte, Liguria and Valle d’Aosta, 10154 Torino, Italy; Paola.Modesto@izsto.it; 4 Department of Internal Medicine and Centre of Excellence for Biomedical Research, University of Genova, 16132 Genova, Italy

**Keywords:** neuroblastoma, fendiline hydrochloride, acetazolamide, drug repositioning, CAIX, non-coding RNAs

## Abstract

High-risk neuroblastoma (HR-NB) still remains the most dangerous tumor in early childhood. For this reason, the identification of new therapeutic approaches is of fundamental importance. Recently, we combined the conventional pharmacological approach to NB, represented by cisplatin, with fendiline hydrochloride, an inhibitor of several transporters involved in multidrug resistance of cancer cells, which demonstrated an enhancement of the ability of cisplatin to induce apoptosis. In this work, we co-administrated acetazolamide, a carbonic anhydrase isoform IX (CAIX) inhibitor which was reported to increase chemotherapy efficacy in various cancer types, to the cisplatin/fendiline approach in SKNBE2 xenografts in NOD-SCID mice with the aim of identifying a novel and more effective treatment. We observed that the combination of the three drugs increases more than twelvefold the differences in the cytotoxic activity of cisplatin alone, leading to a remarkable decrease of the expression of malignancy markers. Our conclusion is that this approach, based on three FDA-approved drugs, may constitute an appropriate improvement of the pharmacological approach to HR-NB.

## 1. Introduction

Neuroblastoma (NB) is a solid early childhood tumor that originates from neural crest tissue [1]. It is one of the most common pediatric solid tumors characterized by heterogeneous clinical traits ranging from disease dissemination to spontaneous healing [2]. More than 90% of patients develop the curable, low risk form of disease, but for patients with high-risk neuroblastoma (HR-NB), less than 50% have a positive outcome [3]. In this scenario new, more effective therapies for NB treatment are urgently needed. Repurposing drugs already used for other pathologies as anticancer agents is a suitable approach to developing new therapeutic strategies in a relatively short time, as molecular targeted drugs already approved for pediatric use could be repurposed thus avoiding phase 1 trials [4,5,6,7,8].

Recently, we proposed a possible alternative pharmacological treatment for HR-NB based on the co-administration of the cytotoxic drug cisplatin and the antianginal agent fendiline hydrochloride, whose synergism increases the effectiveness of anticancer treatment, slowing down tumor growth in a NOD-SCID mouse model of the disease [7]. We found that the beneficial effect of fendiline co-administration is due to its ability to induce the expression of NDM29, an RNA polymerase (pol) III-transcribed non-coding (nc) RNA whose expression induces NB cells to differentiate into a nonmalignant neuron-like phenotype [9]. We demonstrated that NDM29 expression leads to the downregulation of several ABC transporters and an increase of cisplatin retention inside tumor cells and cytotoxicity. Meanwhile in a subsequent work, we also reported a set of nine genes whose inhibition could represent a therapeutic opportunity for HR-NB [10]. Among them, we focused on carbonic anhydrase IX (CAIX) as a possible target for anticancer treatment. Carbonic anhydrases are an ample family of zinc metalloenzymes that catalyze the reversible hydration of carbon dioxide and are strongly induced by hypoxia [11]. CAIX plays a crucial role in the survival strategies adopted by many solid tumors in hypoxic conditions [12]. The expression of CAIX is upregulated in the peri-necrotic areas of poorly vascularized tumors by activating hypoxia inducible factor 1 (HIF1). Often, CAIX expression is detectable only in the tumor cells and not in the surrounding normal tissue, thus representing a marker of cancer development. The activity of CAIX, together with lactate production, renders acidification of the tumor microenvironment, fostering cell detachment from the extracellular matrix and migration to metastatic sites. Another important consequence of tumor microenvironment acidification is the reduced activity of cytotoxic drugs, such as doxorubicin and paclitaxel, since their ionized cell fraction, which is unable to enter the cells, is increased at a low pH [13,14,15]. CAIX overexpression fosters cancer relapse, invasiveness and predicts poor prognosis of malignancies [12,16,17,18].

Compared with normal tissues, CAIX overexpression has been described in a wide spectrum of tumors [19], such as metastatic bladder cancer [20], colon cancer [21], breast cancer [22], and bronchial carcinoid [23]. In relation to our research, the presence of CAIX has also been detected in neuroblastoma and its level of expression is associated with a high proliferation rate, chromosomal deletion 1p, and amplification of oncogene *MYCN*, as well as predicting a poorer prognosis of HR-NB [18,24]. Interestingly, in addition to its use for heart failure, angioedema and glaucoma, acetazolamide, a pharmacological inhibitor of CAIX activity, is currently being evaluated in clinical trials as an adjuvant treatment together with temozolomide in patients with malignant astrocytoma (NIH Clinical Trial.gov https://clinicaltrials.gov/ct2/show/NCT03011671, accessed on 20 January 2019) and in combination with platinum and etoposide in patients with localized small cell lung cancer (https://clinicaltrials.gov/ct2/show/NCT03467360, accessed on 20 December 2020).

Using an in vivo xenograft model, we previously demonstrated that the combined treatment of cisplatin and fendiline reduces NB nodule growth more efficiently than cisplatin alone, thus increasing the overall survival of treated mice [7].

In this report, we evaluate the possible synergism generated by a tri-therapy, adding the CAIX inhibitor acetazolamide to the cisplatin and fendiline hydrochloride combination (Cis/Fen). Indeed, although the effects of carbonic anhydrase inhibition on tumor growth are known, we are particularly interested in verifying whether acetazolamide can further enhance the effects of the combined chemotherapy of HR-NB with Cis/Fen.

## 2. Results

### 2.1. The Co-Administration of Acetazolamide with Cis/Fen Therapy Increased the Mice’s Survival and Inhibits Tumor Nodule Growth

In order to investigate whether acetazolamide enhances the therapeutic efficacy of cisplatin and/or cisplatin/fendiline, we assessed the combined activity of these drugs on tumor growth in vivo, treating NOD-SCID mice which had been subcutaneously xenografted with SKNBE2 NB cells, and evaluated tumor growth rate and animal survival.

The mice were divided into different experimental groups treated with: DMSO 1% in NaCl saline solution 0.9%, as vehicle control; cisplatin (5 mg/kg, Cis); acetazolamide (20 mg/Kg, AZ); fendiline hydrochloride (3 mg/kg, Fen); cisplatin in co-administration with acetazolamide (Cis/AZ); cisplatin in co-administration with fendiline hydrochloride (3 mg/kg, Cis/Fen); cisplatin in co-administration with fendiline hydrochloride and acetazolamide (Cis/Fen/Az).

**Figure 1:**
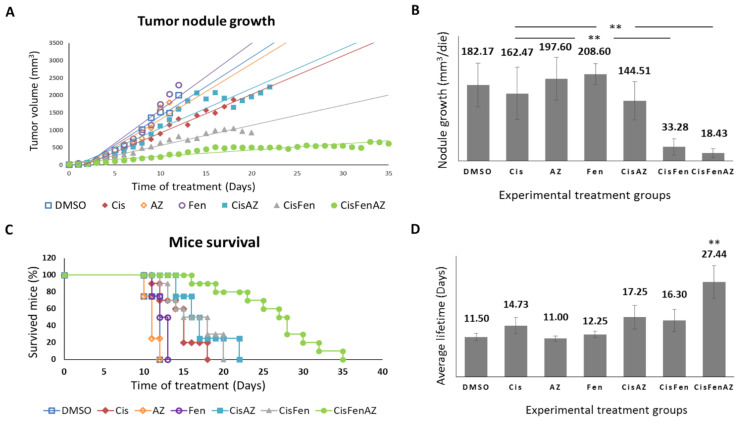
Tumor nodule growth and mice survival. Once SKNBE2 mass reached 5 mm in diameter, mice were treated with DMSO, Cisplatin 5 mg/kg (Cis); acetazolamide 20 mg/Kg (AZ); fendiline hydrochloride 3 mg/kg (Fen); cisplatin 5 mg/kg in co-administration with acetazolamide 20 mg/Kg (CisAZ); cisplatin 5 mg/kg in co-administration with fendiline hydrochloride 3 mg/kg (CisFen); cisplatin 5 mg/kg in co-administration with fendiline hydrochloride 3 mg/kg and acetazolamide 20 mg/Kg (CisFenAZ). The graphs depict linear trend lines of nodule tumor volume (**A**) and average growth of the nodule per day (**B**) in each experimental group. In (**C**), the Kaplan–Meier plot indicates the percentages of mice survival at increasing days after the beginning of treatments. A highly statistically significant decrease of mass growth is induced by CisFen and CisFenAZ treatments (** *p* < 0.001). The histograms in (**D**) represent the average lifetime of mice for each treatment group. A highly statistically significant increase of survival is induced by CisFenAZ (** *p* < 0.001). To compare differences between experimental groups, statistical analysis was performed using one-way analysis of variance (ANOVA) followed by Tukey post hoc test.

Figure 1 reports the growth rate of tumor nodules and mice survival in the different experimental groups. Single treatments with acetazolamide or fendiline per se do not reduce tumor nodule growth, as shown by the daily recordings of nodule volume, which did not differ from DMSO-treated controls; cisplatin alone, at the dose used, had a moderate effect, but, in line with our previous results [7], the combined cisplatin/fendiline treatment reduced tumor growth much more effectively than low-dose cisplatin-based chemotherapy; conversely, no enhancing effects were observed in the co-treatment with cisplatin and acetazolamide, which induced an antitumor effect comparable to cisplatin alone. Interestingly, in the mice treated with the combination therapy which included all three molecules (Cis/Fen/Az), there was a reduced nodule growth rate equal to approximately one tenth of that observed in the vehicle-treated control mice, and if compared with the Cis/Fen treatment, the growth rate halved (Figure 1A,B). Moreover, it was observed that the disease was stable in these mice for up to 35 days, suggesting a sustained activity of the triple-drug combination. Other groups were sacrificed upon reaching the established 2.2 mm^3^ mass volume threshold of the nodule.

In addition, the survival of Cis/Fen/Az-treated mice was prolonged compared to all the other experimental groups. Kaplan–Meier survival curves were analyzed using the Logrank test. Cis/Fen/Az-treated mice showed a highly significant difference in survival when compared to all other groups, on average they survived 27.44 days (*p* < 0.001), whereas Cis/Fen- and Cis/Az-treated mice registered an average survival of 16.3 and 17.25 days respectively, thus only slightly longer and with no statistical significance when compared to treatment with cisplatin alone (14.7 days) (Figure 1C,D). Altogether, these results demonstrate that acetazolamide might ameliorate the chemotherapeutic approach for HR-NB, enhancing the antiblastic effects of low-dose cisplatin and constituting a possible novel anticancer agent to be administered in combination with cisplatin and fendiline hydrochloride.

In addition, in order to clarify whether the action of acetazolamide was restricted to the type of neuroblastoma cell line we used, we verified the same additive effect in vitro on two different lines (IMR32 and SH-SY5Y) and obtained the same effects (see Figure S1).

### 2.2. Acetazolamide in Combination with Cisplatin and Fendiline Hydrochloride Reduces the Expression of Malignancy Markers of NB Xenografts In Vivo

To better characterize the mechanisms of the additive activity of acetazolamide on cisplatin/fendiline treatment, we analyzed the expression of specific markers of cell degeneration and malignancy in the SKNBE2 NB-generated subcutaneous nodules derived from all the above-described treatment groups.

**Figure 2:**
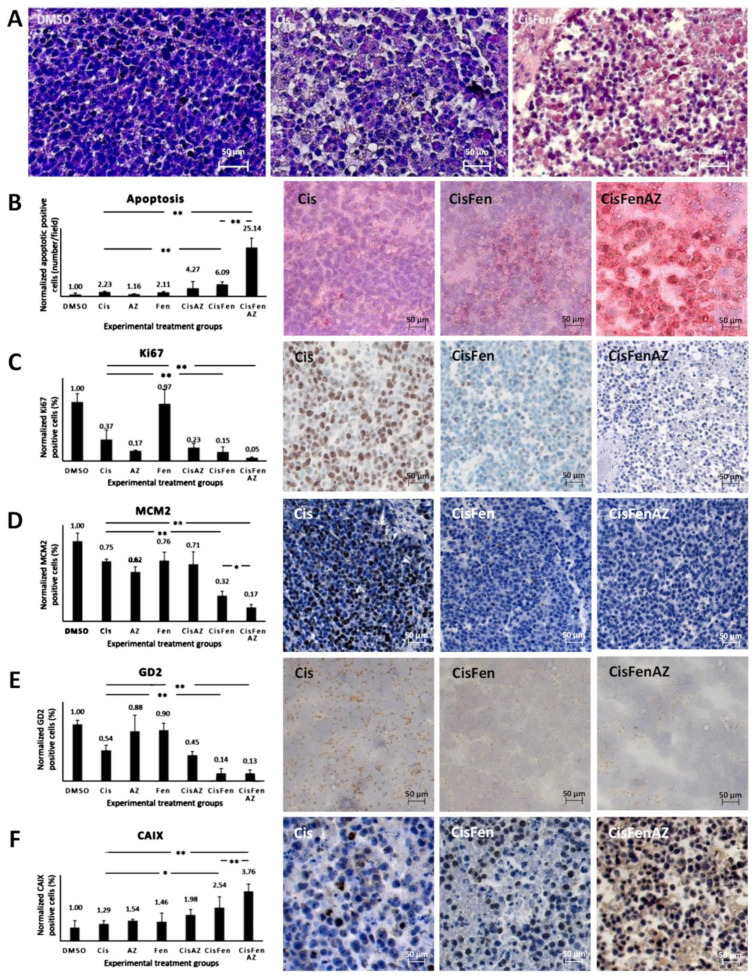
Hystochemical analysis of SKNBE2 nodules. Representative images of hematoxylin staining of SKNBE2 nodules (**A**) from vehicle-(DMSO), cisplatin-(Cis) and cisplatin, fendiline, and acetazolamide-(CisFenAZ) treated mice. The percentage of pycnotic nuclei and mass disaggregation is strongly increased by CisFenAZ treatment. Quantification of apoptotic cells by TUNEL assay in SKNBE2 nodules after treatments (**B**). Values refer to the amount of TUNEL-positive cells per field (left plot). Panels on the right show representative images of cisplatin-(Cis), cisplatin/fendiline-(CisFen) and cisplatin/fendiline/acetazolamide-(CisFenAZ) treated mice. The number of apoptotic cells in cisplatin-treated tumors was increased by the addition of either acetazolamide (4.27 vs. 2.23) or fendiline (6.09 vs. 2.23); remarkably, the contemporary administration of acetazolamide and fendiline with cisplatin produced a strong increase of apoptotic cells (25.14 vs. 2.23). Analysis of Ki-67 (**C**), MCM_2_ (**D**), GD2 (**E**), and CAIX (**F**) expression in SKNBE2 nodules. Plots on the left quantify the amount of positive cells per field. Panels on the right show representative images of cisplatin-(Cis), cisplatin/fendiline-(Cis/Fen) and cisplatin/fendiline/acetazolamide-(CisFenAZ) treated mice. Cisplatin produced a significant reduction of Ki-67, MCM_2_, and GD2. The expression of Ki-67 was strongly reduced by acetazolamide alone and almost abolished when acetazolamide was added along with fendiline and cisplatin (**C**). MCM_2_ reduction by cisplatin was strengthened by fendiline and strengthened further by fendiline/acetazolamide (**D**). The effect of cisplatin on reducing GD2 was strengthened by fendiline only (**E**). All treatments except DMSO produced a significant increase in CAIX expression; the addition of acetazolamide induced a further highly significant CAIX increase, compared with CisFen (* *p* < 0.05, ** *p* < 0.001). Space bar—50 µM.

As shown by hematoxylin staining in Figure 2A, tumors from mice treated with Cis/Fen/Az showed several shrunken cells, with pyknotic nuclei and a less compact framework; nodule excision and manipulation were difficult as they were inconsistent, torn, disrupted, and prone to release a large quantity of dead cells. No viable cell culture was obtained from the disaggregation of the nodules.

The toxicity of the treatments on tumor cells was initially highlighted (Figure 2B, right panels), and quantified (Figure 2B, left plot) by TUNEL assay, to detect apoptosis.

Results showed that: (1) treating the tumor with cisplatin increases the number of apoptotic cells in the nodules by 2.23-fold compared to the DMSO vehicle; (2) treatments with Cis/Az and Cis/Fen increase apoptosis by about 4.27 and 6.09-fold vis-à-vis controls, respectively; (3) the apoptotic rate in the nodules treated with Cis/Fen/Az increases by up to 25-fold. These data show that the addition of Az increases the cytotoxic activity of the cisplatin/fendiline combination by more than 4-fold, indicating an enhancement of the apoptosis rate induced by acetazolamide on the effect of Cis/Fen treatment.

The improved cisplatin/fendiline inhibition of nodule growth caused by acetazolamide (Figure 1B) also suggests that a strong inhibition of cell proliferation may occur. Thus using immunohistochemistry we analyzed the relative percentage of cells of the excised nodules that expressed Ki67 and mini-chromosome maintenance protein 2 (MCM2), a protein involved in the initiation of genome replication now considered a sensitive proliferation marker in several types of human malignancy (Figure 2C,D) [25]. Cisplatin inhibited Ki67-expressing cells to 36% of the vehicle-treated control tumors, the addition of fendiline to cisplatin produced a further reduction to 15.17%, whereas the combination of Cis/Fen/Az leads to the almost complete suppression of expression with a further reduction of Ki67 positive cells (5.23% of DMSO controls) (Figure 2C). Remarkably, the number of MCM2-positive cells was reduced with a similar pattern among treatments than observed for Ki67 (Figure 2D), indicating that the combination of the three drugs is the most effective combination to counteract the proliferation of NB cells in the nodules, with a better outcome than cisplatin and Cis/Fen.

Next, we assessed whether the addition of acetazolamide to previous treatments induced the specific/preferential targeting of the most malignant cells. GD2 is a disialoganglioside antigen whose expression is associated with neuroectodermal origin tumor cells, and is considered a marker of NB malignant cells [26]. We show that GD2-positive cells in sections from both Cis/Fen- and Cis/Fen/Az-treated mice were reduced to a similar level but the presence of these cells was significantly lower (*p* < 0.01) than in nodules from cisplatin-treated mice, and from DMSO-treated control animals (Figure 2E).

Last, we analyzed the possible variation of the number of cells expressing CAIX in response to the inhibition of its activity by acetazolamide. We demonstrate that the synthesis of CAIX in the nodule cells increases along with the efficacy of the therapy, suggesting a likely response to the induction of hypoxia within the nodules caused by cytotoxic treatments. Additionally, in this case the treatment with Cis/Fen was more effective than that with Cis alone (2.53-fold vs. 1.29-fold increase), but the treatment with Cis/Fen/Az led to a 3.76-fold increase, emphasizing once again the effectiveness of acetazolamide as a component of the therapy (Figure 2F) (the statistical analysis of data is reported in Table S1).

## 3. Discussion

HR-NB causes about 15% of deaths from childhood cancers due to frequent relapses, metastasis, and resistance to chemotherapy [27], which is likely to be dependent on stem-like cell populations [28]. In particular, since HR-NB is poorly responsive to the currently available chemo- and radiotherapy protocols [2], innovative treatment approaches are urgently needed. The conventional pharmacological approach to NB is based on cisplatin, either alone or in combination with other chemotherapeutics and radiotherapy [29,30,31,32]. Nevertheless, a high level of resistance occurs through the stable hyperactivation of oncogenes, as *mycN* induces the expression of multidrug resistance genes [33,34].

Increased knowledge of the molecular events leading to tumor growth, self-renewal, invasiveness, and drug resistance, has revealed a number of unexpected pathogens which can be targeted by new and old drugs, in repurposing approaches [4,35]. Remarkably, several of these drugs have already been approved for human therapy, although not for anticancer purposes, and are well tolerated [36]. As a consequence, their repositioning for anticancer therapies is a real opportunity to improve the efficacy of classical cytotoxic drugs. In this context, many studies have also shown the usefulness of drugs that are not directly cytotoxic but are able to counteract the self-defensive machinery of cancer cells, increasing the efficacy of classic chemotherapeutic agents [14].

CAIX inhibition, by acetazolamide, a mild diuretic drug used for heart failure and glaucoma, or related compounds, was reported to inhibit neuroblastoma cell proliferation in conditions of hypoxia, and to increase the efficacy of chemotherapy [24,37]. The additive activity of acetazolamide in combination with histone deacetylase inhibitor MS-275, reduced neuroblastoma cell growth and tumorigenicity, indicating that blocking CAIX activity can improve the outcome when used in combination with both classical and unconventional anticancer drugs [38,39].

It was also reported that fendiline, an L-type calcium blocker, causes cell death and reduction of proliferation and sensitization to cytotoxic drug activity of different tumor cell lines, through different mechanisms [40,41,42,43]. Among those, we recently identified a ncRNA named NDM29 whose stable overexpression is sufficient to induce neuronal differentiation of SKNBE2 NB cells preventing their tumorigenicity in vivo [6,7,9]. NDM29 acts mainly by downregulating the activity of ABC transporters thus increasing the susceptibility of NB cells to the effects of cisplatin. We reported that fendiline induced the expression of NDM29 in SKNBE2 cells to enhance cisplatin toxicity [7]. Given the role of CAIX in protecting tumor mass from hypoxia, we tested the possibility that, similarly to fendiline, acetazolamide might enhance cisplatin efficacy against HR-NB. We show that the antitumor efficacy of cisplatin combined with fendiline on SKNBE2 growth in NOD/SCID mice significantly increased in the presence of acetazolamide, using mass growth and percent of apoptosis and mice survival as parameters of pharmacological treatment efficacy.

The analysis of tumor masses was important to support our previous data about the activity of fendiline [7], since its addition to cisplatin significantly reduced the expression of Ki-67, MCM2, and GD2. Interestingly, acetazolamide, which was ineffective when administered alone and only slightly additive to cisplatin effects, significantly increased Cis/Fen activity as far as all the malignancy parameters analyzed were concerned. In particular, we observed a high induction of apoptosis within tumoral nodules (Figure 2B), which was maximally evident when both acetazolamide and fendiline were added to cisplatin: individually these drugs increased cisplatin proapoptotic activity by two and three-fold, respectively, whereas their use in combination produced a 12.5-fold increase. This evidence argues in favor of the existence of two separate pathways underlying the additive activity of fendiline and fendiline + acetazolamide to cisplatin.

Understanding the mechanisms by which acetazolamide may strengthen the activity of cisplatin is complex since acetazolamide impairs the viability of several tumor cell types acting on distinct pathways independent from the inhibition of CAIX. For example, direct antiproliferative and proapoptotic activity has been observed on laryngeal carcinoma cells through the downregulation of aquaporin1, suggesting that tumor-specific molecules can be acetazolamide-sensitive targets [44]. However, in our experimental model acetazolamide alone did not affect SKNBE2 tumor growth in vivo. The analysis of CAIX expression in SKNBE2 masses showed that the combination of cisplatin and fendiline is stimulated and further increased by the addition of acetazolamide. Our data coincide with previous reports that correlate the expression of CAIX with HR-NB cell survival under hypoxic conditions and the acquisition of multidrug resistance, resulting in worse prognoses [18,24,45]. However, the exposure of neuroblastoma cells to hypoxic conditions in vitro also significantly increases sensitivity to the proapoptotic effects of CAIX inhibitors [24]. In our experimental conditions, SKNBE2 tumor treatment with cisplatin/fendiline produces a hypoxic environment within tumor masses in which cells may survive in the presence of active (or hyperactive) CAIX. The addition of acetazolamide blocks this therapy escape mechanism resulting in an increased antitumor activity. Although promising, the experiments reported here revealed animals suffering after treatment, as the exact relationships between drugs and the different reciprocal dosages still requires a further preclinical phase of development.

In conclusion, the present pilot study provides important proof of principle about the potential synergism of a combination therapy in which acetazolamide is added to fendiline and cisplatin, to increase chemotherapy cytotoxicity. Thus, the repurposing of this commonly used drug may increase the activity of anticancer drugs when conventional treatments are insufficient. However, although acetazolamide is generally well tolerated when administrated in different pathologies, possible side effects of its administration in this combination will need to be investigated in depth in studies analyzing higher number of mice and in more expanded preclinical studies.

## 4. Materials and Methods

### 4.1. Animals

Homozygous male and female NOD-SCID mice were purchased from the Animal Facility of the National Institute for Cancer Research (IST, Genova, Italy). Mice aged about eight weeks were used in the experiments. Animal housing was carried out by the animal facility of the IRCCS Policlinico San Martino (Genoa, Italy). Mice were housed in standard laboratory conditions: 22–24 °C with 12 h light/dark alternation. Water and standard food for mice were available ad libitum. During the experimental procedure, efforts were made to minimize animal stress or discomfort and experiments were carried out in accordance with EU Directive 2010/63/EU. The experimental procedures involving animals were carried out in accordance with the guidelines of the European Community for the use and care of live animals, approved by the Italian Ministry of Health (D.lgs.vo 116/92) and by the Ethics Committee of the Animal Facility of IRCCS Policlinico San Martino (protocol DGSAF 0001448-A).

### 4.2. Cell Cultures

SKNBE2 and SHSY5Y neuroblastoma cells were provided by the cell bank of the National Institute of Cancer Research (IST) Genoa, Italy and obtained from ECACC. SKNBE2 (KCB Cat# KCB 201199YJ, RRID:CVCL_0528) were cultured in RPMI 1640 medium (EUROCLONE S.p.A. Pero, Milan, Italy), supplemented with 10% FBS (EUROCLONE S.p.A. Pero, Milan, Italy), L-glutamine (2 mM; EUROCLONE S.p.A. Pero, Milan, Italy), and penicillin-streptomycin (100 U/mL/100 ug/mL; EUROCLONE S.p.A. Pero, Milan, Italy). SHSY5Y (KCB Cat# KCB 2006107YJ, RRID:CVCL_0019) cells were cultured in Dulbecco’s modified Eagles medium (DMEM) (EUROCLONE S.p.A. Pero, Milan, Italy), 10% FBS (EUROCLONE S.p.A. Pero, Milan, Italy), 2 mM L-glutamine (EUROCLONE S.p.A. Pero, Milan, Italy), and 100 U/mL penicillin-streptomycin (EUROCLONE S.p.A. Pero, Milan, Italy). Cultures were maintained at 37 °C in a humidified 5% CO_2_ atmosphere.

### 4.3. In Vivo Tumor Formation

A total of 3 × 10^6^ SKNBE2 cells were harvested in a serum-free RPMI medium and subcutaneously injected into the flank of NOD/SCID mice. Mice were observed daily to monitor the appearance of tumors at the injection sites. Subsequently, mice were selected randomly to form seven groups and were treated as follows: dimethyl sulfoxide 1% in 0.9% NaCl saline solution (control group, 4 mice); cisplatin 5 mg/kg/dose (10 mice); acetazolamide 20 mg/Kg/dose (4 mice); fendiline hydrochloride 3 mg/kg/dose (8 mice); cisplatin 5 mg/kg/dose in co-administration with acetazolamide 20 mg/Kg/dose (4 mice); cisplatin 5 mg/kg/dose in co-administration with fendiline hydrochloride 3mg/kg/dose (10 mice); cisplatin 5 mg/kg/dose in co-administration with fendiline hydrochloride 3 mg/kg/dose and acetazolamide 20 mg/Kg/dose (10 mice). The groups received dimethyl sulfoxide (DMSO), acetazolamide and fendiline twice a day: once intraperitoneally and once intragastrically. Only cisplatin was injected intraperitoneally once a week. Treatments began when the subcutaneous tumor mass reached a threshold diameter of 5 mm. Tumor size was measured every two days using digital calipers and tumor volume was calculated using the following formula: length^2^ × width × π/6. When the smallest tumor volume reached 2.2 cm^3^, the mice were euthanized by means of CO_2_ narcosis. The mass growth rate was calculated with the following formula: tumor volume at sacrifice (2.2 cm^3^ or more) minus initial volume (when the tumor mass reached the threshold diameter of 5 mm)/number of days to reach the threshold. 

### 4.4. Immunohistochemistry

After the mice were euthanized with CO_2_, the tumor mass was surgically removed, weighed, and measured. The mass was divided into sections and a section was fixed in 4% neutral-buffered formalin, dehydrated, and embedded in paraffin using standard histologic techniques, while a second section was instantly frozen with liquid nitrogen and stored at −80 °C. Immunohistochemistry was performed on 5 μm thick slices. Antigen retrieval was carried out in a citrate buffer (pH 6) for 9 min in a microwave oven. Slices were treated with 3.6% hydrogen peroxide for 10 min to eliminate the endogenous peroxidase activity. Slices were incubated with the following antibodies: anti-Carbonic anhydrase IX mouse antibody (Novus biological, Cambridge, UK, Cat# NBP1-51691, RRID:AB_11011250) diluted 1:400; anti-MCM2 mouse antibody (Thermo scientific, Rockford, IL, USA, Cat#MA5-15895, RRID:AB_11155194) 1:500; anti-Ki-67 rabbit antibody (Novus biological, Abingdon, UK, Cat# NB600-1252, RRID:AB_2142376) 1:200. The reactivity to GD2 was assessed by using a section of frozen tissue embedded in OCT (Killik, Bio-Optica, Milano, Italy), cooled by liquid nitrogen and cut into 5 µm sections by a cryostat at −20 °C. The slices were allowed to dry for 24 h and briefly fixed in paraformaldehyde vapor for 5 min. The sections were then stained using mouse anti-human disialoganglioside GD2 monoclonal antibody (Millipore, Temecula, CA, USA, Cat# MAB2052, RRID:AB_11212189) at a dilution of 1:200. For negative controls, the primary antibody was omitted. The immunoreactions were visualized using 3,3′-diaminobenzidine (DAB) chromogen (Dako—Glostrup, Denmark). The slices were counterstained with Harris hematoxylin (Applichem Panreac, Darmstadt, Germany). The TUNEL assay was performed using the in situ cell death detection kit, AP (Roche Diagnostic GmbH, Mannheim, Germany) following the manufacturer’s instructions. After the Tunel reaction, the chromogen used was Fast-Red Substrate System (Thermo Scientific, Fremont, CA, USA) following the manufacturer’s instructions. Tissue slides were observed and scanned using Aperio AT2—Scanner (Leica Microsystems Srl, Milano, Italy) at ×20 magnification. After capturing eight random fields of each image per each mouse, the positive area in slices was quantified using ImageJ software downloaded from the NIH website (http://rsb.info.nih.gov/ij, RRID:SCR_003070, accessed on 20 December 2019) [46]. Only Ki-67 quantification was performed using ImmunoRatio (http://153.1.200.58:8080/immunoratio/, accessed on 20 December 2019) [47]. The value assigned to each tumor was obtained from the average of the values of single fields. The value of each group represents the mean of the values obtained from the mice treated in the same way.

### 4.5. Statistical Analysis

The mean and standard deviations were calculated for each group, and a one-way ANOVA (analysis of variance) with post hoc Tukey HSD (honestly significant difference) test was used to determine significance between the groups analyzed (*p* values < 0.05) with a 95% confidence interval (http://astatsa.com/OneWay_Anova_with_TukeyHSD/, accessed on 10 January 2020). The data are presented as mean ± SD. In graphs, statistical significance is displayed as * *p* < 0.05, and ** *p* < 0.01. The difference in mice survival between groups was calculated with a Logrank test performed using an online calculator (https://www.evanmiller.org/ab-testing/survival-curves.html, accessed on 10 January 2020).

## Supplementary Materials

The following are available online at https://www.mdpi.com/article/10.3390/ijms22136753/s1.
