# Supplementary material for: Efficacy of a Three Drug-Based Therapy for Neuroblastoma in Mice
Source: Int J Mol Sci. 2021 Jun 23;22(13):6753. doi: 10.3390/ijms22136753 (PMC8268736; doi:10.3390/ijms22136753)
Supplement: Supplementary file 1 [file ijms-22-06753-s001.zip › Table S1.pptx]

## Slide 1
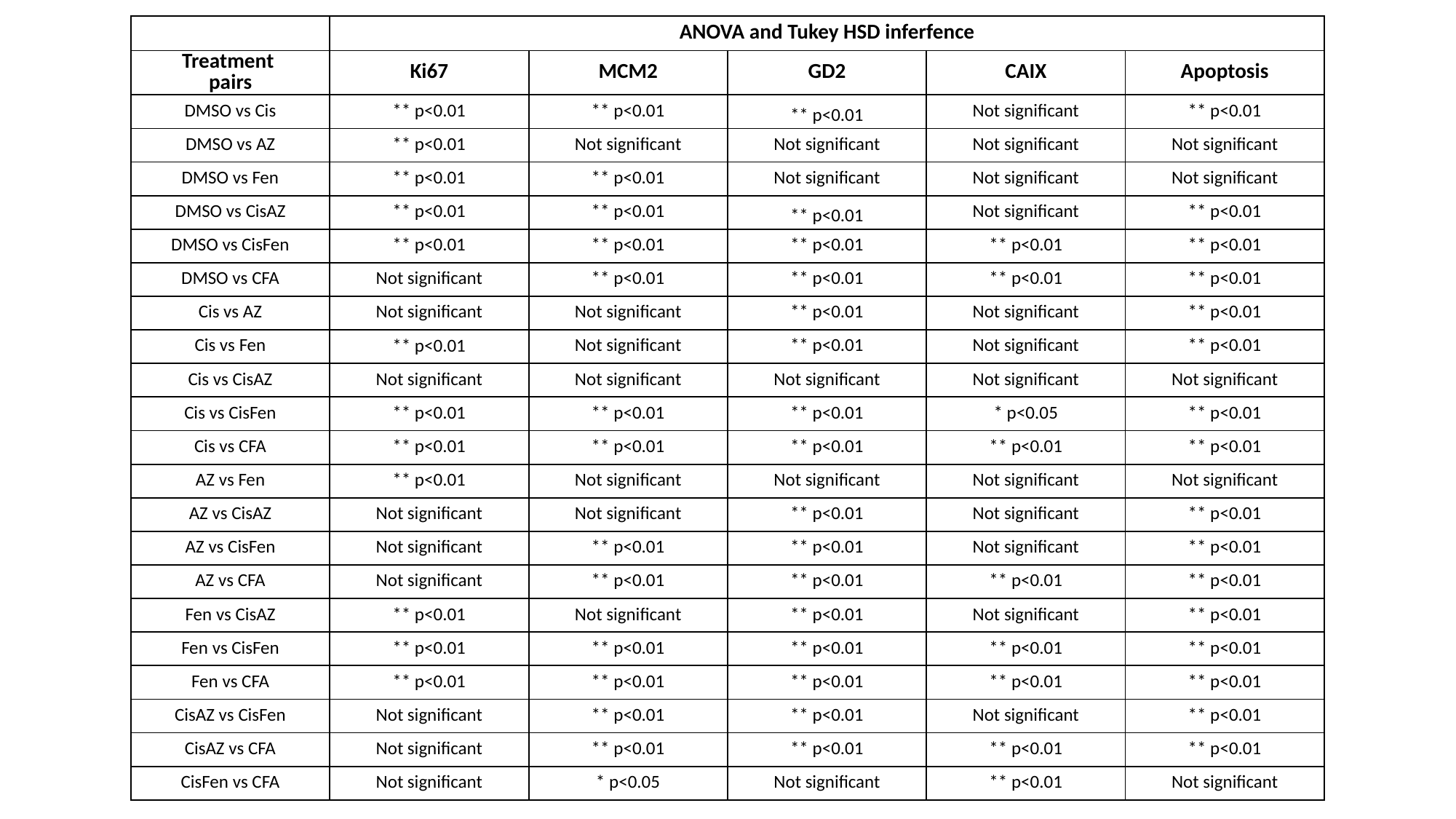

| | ANOVA and Tukey HSD inferfence | | | | |
| --- | --- | --- | --- | --- | --- |
| Treatment  pairs | Ki67 | MCM2 | GD2 | CAIX | Apoptosis |
| DMSO vs Cis | \*\* p<0.01 | \*\* p<0.01 | \*\* p<0.01 | Not significant | \*\* p<0.01 |
| DMSO vs AZ | \*\* p<0.01 | Not significant | Not significant | Not significant | Not significant |
| DMSO vs Fen | \*\* p<0.01 | \*\* p<0.01 | Not significant | Not significant | Not significant |
| DMSO vs CisAZ | \*\* p<0.01 | \*\* p<0.01 | \*\* p<0.01 | Not significant | \*\* p<0.01 |
| DMSO vs CisFen | \*\* p<0.01 | \*\* p<0.01 | \*\* p<0.01 | \*\* p<0.01 | \*\* p<0.01 |
| DMSO vs CFA | Not significant | \*\* p<0.01 | \*\* p<0.01 | \*\* p<0.01 | \*\* p<0.01 |
| Cis vs AZ | Not significant | Not significant | \*\* p<0.01 | Not significant | \*\* p<0.01 |
| Cis vs Fen | \*\* p<0.01 | Not significant | \*\* p<0.01 | Not significant | \*\* p<0.01 |
| Cis vs CisAZ | Not significant | Not significant | Not significant | Not significant | Not significant |
| Cis vs CisFen | \*\* p<0.01 | \*\* p<0.01 | \*\* p<0.01 | \* p<0.05 | \*\* p<0.01 |
| Cis vs CFA | \*\* p<0.01 | \*\* p<0.01 | \*\* p<0.01 | \*\* p<0.01 | \*\* p<0.01 |
| AZ vs Fen | \*\* p<0.01 | Not significant | Not significant | Not significant | Not significant |
| AZ vs CisAZ | Not significant | Not significant | \*\* p<0.01 | Not significant | \*\* p<0.01 |
| AZ vs CisFen | Not significant | \*\* p<0.01 | \*\* p<0.01 | Not significant | \*\* p<0.01 |
| AZ vs CFA | Not significant | \*\* p<0.01 | \*\* p<0.01 | \*\* p<0.01 | \*\* p<0.01 |
| Fen vs CisAZ | \*\* p<0.01 | Not significant | \*\* p<0.01 | Not significant | \*\* p<0.01 |
| Fen vs CisFen | \*\* p<0.01 | \*\* p<0.01 | \*\* p<0.01 | \*\* p<0.01 | \*\* p<0.01 |
| Fen vs CFA | \*\* p<0.01 | \*\* p<0.01 | \*\* p<0.01 | \*\* p<0.01 | \*\* p<0.01 |
| CisAZ vs CisFen | Not significant | \*\* p<0.01 | \*\* p<0.01 | Not significant | \*\* p<0.01 |
| CisAZ vs CFA | Not significant | \*\* p<0.01 | \*\* p<0.01 | \*\* p<0.01 | \*\* p<0.01 |
| CisFen vs CFA | Not significant | \* p<0.05 | Not significant | \*\* p<0.01 | Not significant |
