# Supplementary figures and images for: Efficacy of a Three Drug-Based Therapy for Neuroblastoma in Mice
Source: Int J Mol Sci. 2021 Jun 23;22(13):6753. doi: 10.3390/ijms22136753 (PMC8268736; doi:10.3390/ijms22136753)

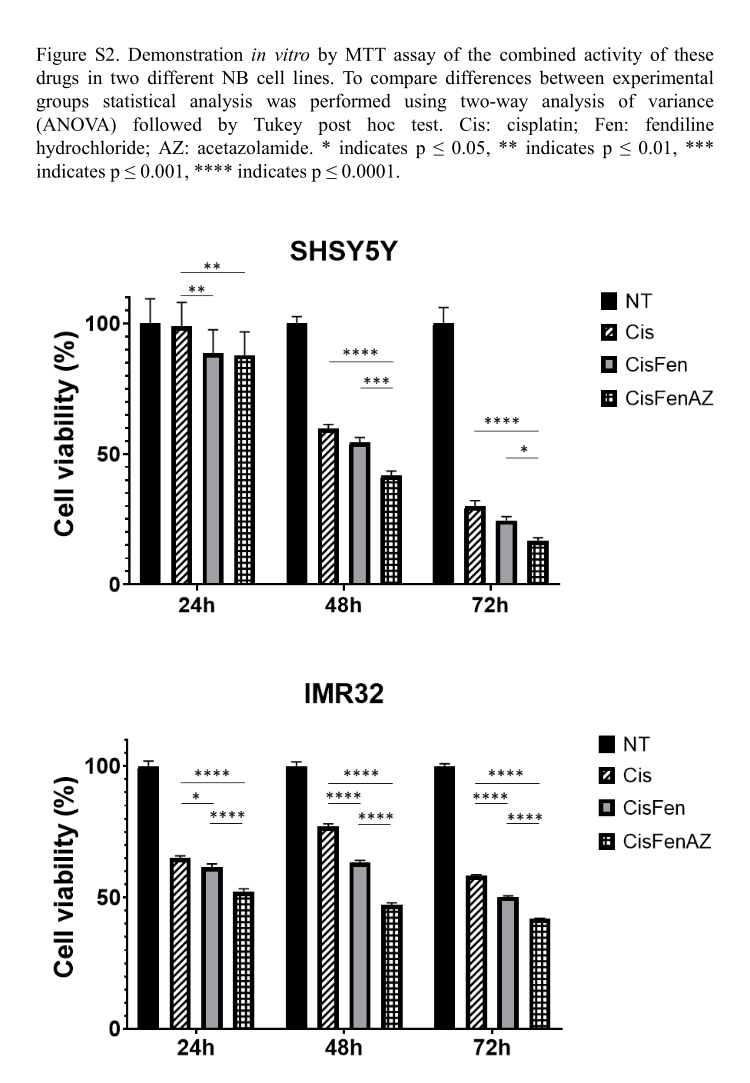

Supplement: Supplementary file 1 [file ijms-22-06753-s001.zip › Figure S1.tif]
